# The Time Trend Temperature–Mortality as a Factor of Uncertainty Analysis of Impacts of Future Heat Waves

**DOI:** 10.1289/ehp.1308042

**Published:** 2014-05-01

**Authors:** Cristina Linares, Isidro J. Mirón, Juan C. Montero, Juan J. Criado-Álvarez, Aurelio Tobías, Julio Díaz

**Affiliations:** 1 National School of Public Health, Carlos III Institute of Health, Madrid, Spain; 2 Torrijos Public Health District, Castile-La Mancha Regional Health & Social Affairs Authority (Consejería de Sanidad y Asuntos Sociales de Castilla-La Mancha), Torrijos (Toledo), Spain; 3 Health Sciences Institute, Castile-La Mancha Regional Health & Social Affairs Authority, Talavera de la Reina (Toledo), Spain; 4 Health Service of Castile-La Mancha (SESCAM), San Bartolomé de las Abiertas (Toledo), Spain; 5 Spanish Council for Scientific Research (Consejo Superior de Investigaciones Científicas; CSIC), Institute of Environmental Assessment and Water Research (Instituto de Diagnóstico Ambiental y Estudios del Agua; IDAEA), Barcelona, Spain.

Recently, the paper by Wu et al. (2014), “Estimation and Uncertainty Analysis of Impacts of Future Heat Waves on Mortality in the Eastern United States,” concluded that “the major sources of uncertainty were the relative risk estimates for mortality on heat wave versus non–heat wave days, the RCP scenarios, and the heat wave definitions.” One conclusion to be drawn from reading this manuscript might be that a good definition of “heat wave” based on epidemiological studies and accurate determination of the risks associated with such temperatures would greatly reduce these uncertainties. Although the authors allude to the possible geographic variability of these risks, there is nevertheless no mention of the possible evolution over time that can take place both in heat-wave definition temperatures and in the modifications of these possible impacts, beyond those stemming from the use of air-conditioning equipment and the implementation of heat-wave prevention plans.

Along these lines, recent studies have found that demographic and socioeconomic factors may be behind the trend in minimum mortality temperatures (Mirón et al. 2008). Hence, in Castile-La Mancha (Spain) the minimum mortality temperature went from 32°C in the decade 1975–1985 to 28°C in 1995–2003 as a consequence of population aging. This fact influences the heat-wave definition temperatures, which are very closely linked to the age group > 65 years (Montero et al. 2012).

Added to this uncertainty are the shifts over time observed in the impact of heat waves. Studies conducted in different parts of the world show that, far from remaining constant, these impacts are changing over time, with a trend toward minimizing such effects (Schifano et al. 2012): Although the effect is most pronounced in cardiovascular mortality (Ha and Kim 2013), it has remained practically constant in the case of respiratory mortality (Mirón et al, in press). These results, obtained from a time series covering > 30 years, show that the increase in risk of heat-related mortality for each degree centigrade that the threshold temperature is exceeded went from 13.7% in 1975–1985 to 7.4% in 1997–2008, and specifically that this decline was attributable to circulatory causes, going from 18.2% in 1975–1985 to 5.8% in 1997–2008. In the case of respiratory causes, however, no such decline was in evidence, with the respective figures remaining practically constant: 11.8% in 1975–1985 versus 13.5% in 1997–2008. This pattern would seem to be linked to improvements in health care services (particularly in the case of patients with cardiovascular diseases), socioeconomic improvements, and the provision of infrastructures for better living conditions. It therefore follows that any changes in the trend of these parameters could reverse the situation and increase the effects of temperature extremes on mortality. This decline in heat wave–related mortality does not appear to be connected with the implementation of prevention plans, in Spain at least (Culqui et al. 2013).

Because the factors that appear to influence the shifts in the relationship between temperature and mortality are not local and are thus extrapolatable to a large proportion of developed countries, their relevance is self-evident.

These uncertainties add to those already cited in the paper by Wu et al. and highlight the need for more in-depth knowledge, not only of temperature forecasts at the different time horizons, but also of the behavior pattern over time of the temperature–mortality relationship. Far from being constant, this relationship displays a time trend that is seldom taken into account in the models used to predict the impact of climate change on human health.
